# Environmental factors, seven GWAS‐identified susceptibility loci, and risk of gastric cancer and its precursors in a Chinese population

**DOI:** 10.1002/cam4.1038

**Published:** 2017-02-21

**Authors:** Meng Cai, Shuyang Dai, Wanqing Chen, Changfa Xia, Lingeng Lu, Shuguang Dai, Jun Qi, Minjie Wang, Meilin Wang, Lanping Zhou, Fuhua Lei, Tingting Zuo, Hongmei Zeng, Xiaohang Zhao

**Affiliations:** ^1^ State Key Laboratory of Molecular OncologyNational Cancer Center/Cancer HospitalChinese Academy of Medical Sciences and Peking Union Medical CollegeBeijingChina; ^2^ National Office for Cancer Prevention and ControlNational Cancer Center/Cancer HospitalChinese Academy of Medical Sciences and Peking Union Medical CollegeBeijingChina; ^3^ Department of Chronic Disease EpidemiologyYale School of Public HealthSchool of MedicineYale Cancer CenterYale UniversityNew Haven, ConnecticutUSA; ^4^ Center for Disease Control and Prevention of Sheyang CountySheyangJiangsuChina; ^5^ Department of Clinical LaboratoryNational Cancer Center/Cancer HospitalChinese Academy of Medical Sciences and Peking Union Medical CollegeBeijingChina; ^6^ Department of Environmental GenomicsJiangsu Key Laboratory of Cancer BiomarkersPrevention and TreatmentCollaborative Innovation Center for Cancer Personalized MedicineNanjing Medical UniversityNanjing, JiangsuChina; ^7^ Department of Genetic Toxicologythe Key Laboratory of Modern Toxicology of Ministry of EducationSchool of Public HealthNanjing Medical UniversityNanjingJiangsuChina; ^8^ Department of PathologyFeicheng People HospitalFeicheng, ShandongChina

**Keywords:** Gene–environment interactions, genetic variant, GWAS, gastric cancer, *Helicobacter pylori*, precancerous lesions

## Abstract

Gene–environment interactions may increase gastric cancer (GC) risk. Seven susceptibility loci identified by genome‐wide association studies (GWASs) suggest that genetic factors play a role in gastric carcinogenesis. Meanwhile, *Helicobacter pylori* (*H. pylori*) infection, smoking, and alcohol drinking are also important environmental factors for gastric cancer. However, studies to explore the role of gene–environment interactions in gastric carcinogenesis, and particularly the relationship between the seven susceptibility loci and their potential interactions with *H. pylori* infection, smoking, and alcohol drinking in risk of GC, and severe intestinal metaplasia (IM)/dysplasia, have been inconclusive. A total of 1273 subjects in a Chinese population were recruited, and genotyping was carried out using the competitive allele‐specific PCR (KASP) method. Unconditional logistic regression was applied to model the associations between genetic polymorphisms and the disease risk. Effect modifications by *H. pylori* infection, smoking and alcohol drinking were evaluated. *PSCA* rs2294008/rs2976392 showed a significant, multiplicative interaction with *H. pylori* infection in risk of GC. Meanwhile, *PRKAA1* rs13361707 had an additive interaction with *H. pylori* infection. *SLC52A3* rs13042395 showed an interaction with alcohol drinking in risk of GC. Moreover, three SNPs, *MUC1* rs4072037, *ZBTB20* rs9841504 and *PRKAA1* rs13361707, were associated with precancerous gastric lesions (severe IM/dysplasia). Our data suggest that genetic predisposition factors identified by GWAS may interact with environmental risk factors, Particularly for *H. pylori* infection and alcohol consumption, to increase the risk of GC.

## Introduction

Gastric cancer (GC) is one of the most common cancers worldwide and more than 40% of GC cases occur in China. In 2015, approximately 500,000 patients in China died of GC 1. GC can be divided into intestinal and diffuse types according to its histopathological features. The carcinogenesis of GC is a multifactorial process, resulting from the combined consequences of genetic predisposition and environmental risk factors including *Helicobacter pylori* (*H. pylori*) infection, smoking, alcohol drinking, and dietary factors 2.

Among the environmental risk factors for GC, it is widely recognized that *H. pylori* is a major etiological factor 3. *Helicobacter pylori*, a Gram‐negative bacterium, presents higher prevalence in developing countries than in developed countries 4. Nevertheless, only a small proportion of *H. pylori*‐infected individuals progress to GC 5, suggesting that environmental factors other than *H. pylori* infection, such as smoking and alcohol drinking, and genetic predisposition may also influence the outcome of gastric pathogenesis 6. Several studies have found that *H. pylori* cytotoxin‐associated gene A (CagA)^+^ and/or vacuolating cytotoxin gene A (VacA)^+^ strains can increase the risk of developing gastric diseases. In particular, Type I strains of *H. pylori* with CagA^+^ and/or VacA^+^ have higher pathogenicity than Type II strains of *H. pylori* without CagA^+^ and VacA^+^ 7. Some in vitro studies have demonstrated that several genes within the *H. pylori* cag islands are involved in the release of proinflammatory cytokines 8, 9. VacA is a secreted protein that stimulates epithelial cell apoptosis and induces vacuole formation in eukaryotic cells 10.

Four genome‐wide association studies (GWASs) have identified seven GC genetic variants, including *MUC1* rs4072037 at 1q22, *ZBTB20* rs9841504 at 3q13, *PRKAA1* rs13361707 at 5p13, *PSCA* rs2976392 and rs2294008 at 8q24, *PLCE1* rs2274223 at 10q23, and *SLC52A3* rs13042395 at 20p13 11, 12, 13, 14, 15. These studies provide important clues to the etiology of the disease. Subjects carrying a certain genetic variant may be at greater risk for GC if a related environmental factor exists. Such gene–environment interactions have been identified in several cancers, including cancers of the bladder, esophagus and stomach 16, 17, 18. Our previous study found that *TLR5* gene polymorphism might be modified by *H. pylori* infection 19. Li et al. 20 revealed that *MUC1* rs4072037, *PRKAA1* rs13361707, and *PLCE1* rs2274223 may interact with *H. pylori* infection and increase the risk of GC. Severe intestinal metaplasia (IM) and dysplasia are precancerous lesions of GC. The risk of GC generally increases with the histological grade (from low to high grade) of IM and dysplasia 21. Many polymorphisms associated with GC may also be related to gastric precancerous lesions 22, 23.

In view of the role of genetic effects and gene–environment interactions in gastric carcinogenesis, we were particularly interested in the seven GWAS‐identified genetic variants and their potential roles in GC and severe IM/dysplasia. We explored the seven susceptibility loci and their potential interactions with *H. pylori* infection, smoking and alcohol drinking in risk of GC and its precursors in a total of 1273 subjects in a Chinese population.

## Materials and Methods

### Study subjects

In 2014, we launched an endoscopic screening program for GC in a cohort from Sheyang County, Jiangsu Province, China. Overall, 2189 subjects were recruited for endoscopic screening of GC. All screened subjects received an endoscopic survey and provided blood samples for DNA isolation and *H. pylori* serological detection. Biopsy samples were taken from the standard locations of the stomach in each subject, and the histopathological diagnosis of each sample was made by senior pathologists. Each biopsy was classified into either normal, nonatrophic gastritis, atrophic gastritis, mild/severe IM, low/high grade dysplasia, or GC based on the Chinese System and the criteria for the Updated Sydney System 24, 25. The TNM stages were evaluated according to the American Joint Committee on Cancer Staging Manual, Sixth Edition. Demographic and epidemiologic information, including age, sex, smoking, alcohol drinking and family history of cancer, was recorded on a questionnaire through face‐to‐face interviews by a trained doctor.

The present study included 501 GC cases from the baseline gastroscopic screening program in Sheyang County of China and from the Cancer Hospital of Chinese Academy of Medical Sciences diagnosed during the same period. Additionally, 489 controls with either normal or nonatrophic gastritis were randomly selected from the cohort and frequency‐matched to the GC cases by gender and age. Moreover, 283 cases with severe IM (31) or dysplasia (252) identified in the gastroscopic screening cohort were included. Ethics approval was obtained from the Institutional Review Board of the Cancer Hospital of the Chinese Academy of Medical Sciences, and all participants provided written informed consent.

### DNA extraction and genotyping

Genomic DNA was extracted from peripheral blood using a whole‐blood DNA kit (Biomed Corporation, China) following the manufacturer's instructions. Isolated genomic DNA was analyzed by 0.8% agarose gel electrophoresis to evaluate DNA quality, and DNA quantity was assessed using a NanoDrop Spectrophotometer (Thermo Scientific, Waltham, MA, USA). The DNA samples were stored at −20°C until use. Genotyping of the seven candidate SNPs was performed by competitive allele‐specific PCR (KASP) assay (Compass Biotechnology, Beijing, China). Detailed information on the genotyping method has been described previously 26, 27. The sequences of the primers used in this study are available upon request. All the primers were synthesized by LGC Genomics (Herts, UK).The call rate for each SNP was >95%. Three percent of the samples were randomly selected for validation via DNA sequencing, and the concordance was 100%.

### *Helicobacter pylori* serology

*Helicobacter pylori* infection status was determined using an immunoblotting assay according to the manufacturer's instructions (Shenzhen Blot Biotech Co., Ltd., Shenzhen, China). The sensitivity and specificity of the assay were 96.8% and 97.6%, which were higher than another similar immunoblotting kit Helicoblot 2.1 (with 95.6% and 92.6% of the sensitivity and specificity) 28, 29, 30. The assay allows the identification of specific antibody responses against the distinct *H. pylori* antigens CagA, VacA, UreA and UreB. Briefly, the lysate from *H. pylori* was separated by SDS‐PAGE and then electrotransferred onto polyvinylidene fluoride membranes and incubated with 1:100 diluted serum for 30 min. After washing, the membrane strips were incubated with horse radish peroxidase‐conjugated anti‐human immune globulin antibodies (IgG, dilution 1:50) for 30 min at room temperature. After washing three times, the bound antibodies were visualized through a staining reaction, and the corresponding antigens formed dark bands on the strip. If such a band formed, the subject was considered seropositive for the specific *H. pylori* antigen. *Helicobacter pylori* infection status was defined as seropositive if any of the four antigens tested, CagA, VacA, UreA or UreB, was positive. Subjects positive for CagA and/or VacA were classified as Type I *H. pylori* seropositive, and those positive for only UreA and/or UreB were classified as Type II *H. pylori* seropositive.

### Statistical analysis

The chi‐square test was used to compare the basic characteristics and the genotypes between cases and controls. Goodness‐of‐fit chi‐square test was used to test the Hardy–Weinberg equilibrium in the controls. Odds ratios (ORs) and their corresponding 95% confidence intervals (CIs) were calculated for the association of genetic variants with risk of GC and severe IM/dysplasia, as well as for gene–gene or gene–environmental factor joint effect analysis using unconditional logistic regression models. All analyses were adjusted for potential confounders including age, sex, *H. pylori* infection, smoking and alcohol drinking. For gene–gene or gene–environment (*H. pylori*, smoking and alcohol drinking) joint effect analysis, different gene–gene or gene–environment combination categories were transformed into dummy variables, with the “except” category used as reference; all the other categories were entered into the unconditional logistic regression model to calculate the adjusted ORs and 95% CIs for each category compared with the reference. Multiplicative gene–gene or gene–environment interactions were measured by including main effect variables and their product terms in the logistic regression model with likelihood ratio tests. For the additive interaction analysis, we used a bootstrapping test of goodness of fit of the null hypothesis of no departure from an additive model versus an alternative hypothesis of a departure from an additive model using Stata (version 12.0; StataCorp LP, College Station, TX). A synergy index (SI) and 95% CI were reported. Linkage disequilibrium (LD) coefficients were calculated with Haploview 4.0 software. All *P* values reported were unadjusted, two‐sided, and considered statistically significant at *P* ≤ 0.05. The Benjamini–Hochberg procedure was performed for multiple test comparison correction, and the false discovery rate (FDR)‐adjusted *P* value was considered as well 31. All the statistical analyses were performed using SPSS (version 16.0).

## Results

### Characteristics of study subjects

A total of 1273 subjects, including 870 males (68.3%) and 403 females (31.7%) were included in our study, and the mean age of the participants was 58.0 years. The baseline characteristics of the study population are shown in Table 1. Of 501 GC cases, 384 (83.8%) occurred at a noncardia site and 74 (16.2%) were at a cardia site. There were no significant differences in the distributions of age and sex between GC cases and controls. However, the percentages of smoking, alcohol drinking, and *H. pylori* infection were significantly higher in the GC cases than in the controls (*P *=* *0.037 for smoking status, *P *<* *0.001 for alcohol drinking and *P *<* *0.001 for *H. pylori* seropositivity). For IM/dysplasia and controls, the distribution of age, smoking and alcohol drinking was similar between groups. However, the percentage of female and *H. pylori* seropositivity were significantly higher in the IM/dysplasia cases than in the controls (*P *<* *0.001 for sex and *P *<* *0.001 for *H. pylori* infection).

**Table 1:** Basic characteristics of the study subjects

	GC cases	Controls	Severe IM/dysplasia
*n*	501	489	283
Age, *n* (%)			
<58	246 (49.1)	233 (47.6)	133 (47.0)
≥58	255 (50.9)	256 (52.4)	150 (53.0)
*P* a	0.647		0.861
Sex, *n* (%)			
Male	365 (72.9)	352 (72.0)	153 (54.1)
Female	136 (27.1)	137 (28.0)	130 (45.9)
*P* a	0.759		<0.001
Smoking, *n* (%)			
Yes	199 (46.9)	196 (40.1)	112 (39.6)
No	225 (53.1)	293 (59.9)	171 (60.4)
*P* a	0.037		0.890
Alcohol drinking, *n* (%)			
Yes	186 (44.3)	119 (24.3)	65 (23.0)
No	234 (55.7)	370 (75.7)	217 (77.0)
*P* a	<0.001		0.687
*H. pylori* infection, *n* (%)			
Positive	431 (88.0)	280 (57.4)	217 (78.3)
Negative	59 (12.0)	208 (42.6)	60 (21.7)
*P* a	<0.001		<0.001
Type of *H. pylori* infection, *n* (%)			
CagA^+^	322 (65.7)	158 (32.4)	115 (41.5)
CagA^−^	168 (34.3)	330 (67.6)	162 (58.5)
*P* a	<0.001		0.011
Type of *H. pylori* infection, *n* (%)			
VacA^+^	228 (46.5)	137 (28.1)	85 (30.7)
VacA^−^	262 (53.5)	351 (71.9)	192 (69.3)
*P* a	<0.001		0.444
Type of *H. pylori* infection, *n* (%)			
Type I	329 (67.1)	163 (33.4)	133 (48.0)
Type II	102 (20.8)	117 (24.0)	84 (30.3)
Not infected	59 (12.1)	208 (42.6)	60 (21.7)
*P* a	<0.001		<0.001
Cancer site, *n* (%)			
Cardia	74 (16.2)		
Noncardia	384 (83.8)		
Stage, *n* (%)			
Stage I/II	98 (25.8)		
Stage III/IV	282 (74.2)		

a *P* values for chi‐square test.

For type of *H. pylori* infection, there was a higher degree of *H. pylori* CagA positivity and VacA positivity in GC patients than in controls (65.7% vs. 32.4% and 46.5% vs. 28.1%). Subjects with Type I *H. pylori* infection had a significantly higher risk of GC compared with those without *H. pylori* infection (OR: 7.12, 95% CI: 5.04–10.04). For subjects with Type II *H. pylori* infection, they had a two–fold increased risk of GC compared to those without *H. pylori* infection.

### Main effects of the seven SNPs with risk of GC

The genotype distribution of the seven SNPs in all subjects is shown in Table 2. The *PSCA* rs2294008 was in 100% LD with rs2976392 (*r* ^2^ = 1.00). Under the dominant models, we found that individuals with at least one variant allele of the six polymorphisms had an altered risk of GC (OR: 1.48, 95% CI: 1.09–1.99 for *PLCE1* rs2274223; OR: 1.41, 95% CI: 1.05–1.89 for *PSCA* rs2294008; OR: 1.41, 95% CI: 1.05–1.89 for *PSCA* rs2976392; OR: 0.59, 95% CI: 0.42–0.81 for *MUC1* rs4072037; OR: 0.70, 95% CI: 0.52–0.93 for *SLC52A3* rs13042395; and OR: 0.43, 95% CI: 0.31–0.61 for *PRKAA1* rs13361707). For *ZBTB20* rs9841504, although subjects carrying the CG genotype had a decreased risk of GC (OR: 0.65, 95% CI: 0.45–0.92), we did not observe a significant association between *ZBTB20* rs9841504 and risk of GC under the dominant model.

**Table 2:** Distribution of genotype frequencies of the susceptibility loci and their associations with GC risk

Genotype	Genetic model	Controls *n* (%)	GC cases *n* (%)	OR (95% CI)^a^	*P*
*PLCE1* rs2274223					
AA	Codominant	317 (65.0)	291 (59.3)	1	
AG		153 (31.3)	174 (35.4)	1.40 (1.03–1.91)	0.033
GG		18 (3.7)	26 (5.3)	2.18 (1.07–4.45)	0.032
AA	Dominant	317 (65.0)	291 (59.3)	1	
AG+GG		171 (35.0)	200 (40.7)	1.48 (1.09–1.99)	0.011
G allele	Additive			1.43 (1.11–1.84)	0.005
*PSCA* rs2294008					
CC	Codominant	268 (54.9)	215 (44.3)	1	
CT		173 (35.5)	225 (46.4)	1.50 (1.10–2.04)	0.01
TT		47 (9.6)	45 (9.3)	1.10 (0.66–1.83)	0.725
CC	Dominant	268 (54.9)	215 (44.3)	1	
CT+TT		220 (45.1)	270 (55.7)	1.41 (1.05–1.89)	0.021
T allele	Additive			1.20 (0.96–1.49)	0.112
*PSCA* rs2976392					
GG	Codominant	268 (54.9)	213 (44.3)	1	
AG		173 (35.5)	223 (46.4)	1.49 (1.10–2.04)	0.011
AA		47 (9.6)	45 (9.3)	1.11 (0.66–1.85)	0.699
GG	Dominant	268 (54.9)	213 (44.3)	1	
AA+AG		220 (45.1)	268 (55.7)	1.41 (1.05–1.89)	0.021
A allele	Additive			1.20 (0.96–1.50)	0.109
*MUC1* rs4072037					
AA	Codominant	318 (65.2)	371 (77.3)	1	
AG		152 (31.1)	92 (19.2)	0.54 (0.38–0.76)	<0.001
GG		18 (3.7)	17 (3.5)	1.01 (0.48–2.15)	0.974
AA	Dominant	318 (65.2)	371 (77.3)	1	
AG+GG		170 (34.8)	109 (22.7)	0.59 (0.42–0.81)	0.001
G allele	Additive			0.71 (0.54–0.93)	<0.001
*ZBTB20* rs9841504					
CC	Codominant	349 (71.5)	368 (75.6)	1	
CG		124 (25.4)	95 (19.5)	0.65 (0.45–0.92)	0.015
GG		15 (3.1)	24 (4.9)	1.85 (0.86–3.97)	0.114
CC	Dominant	349 (71.5)	368 (75.6)	1	
CG+GG		139 (28.5)	119 (24.4)	0.76 (0.54–1.05)	0.094
G allele	Additive			0.91 (0.70–1.19)	0.486
*SLC52A3* rs13042395					
CC	Codominant	180 (36.8)	238 (48.7)	1	
CT		247 (50.5)	211 (43.1)	0.77 (0.56–1.04)	0.088
TT		62 (12.7)	40 (8.2)	0.46 (0.27–0.76)	0.003
CC	Dominant	180 (36.8)	238 (48.7)	1	
CT+TT		309 (63.2)	251 (51.3)	0.70 (0.52–0.93)	0.016
T allele	Additive			0.71 (0.56–0.88)	0.002
*PRKAA1* rs13361707					
CC	Codominant	98 (20.1)	172 (36.4)	1	
CT		246 (50.5)	213 (45.0)	0.48 (0.34–0.68)	<0.001
TT		143 (29.4)	88 (18.6)	0.35 (0.23–0.54)	<0.001
CC	Dominant	98 (20.1)	172 (36.4)	1	
CT+TT		389 (79.9)	301 (63.6)	0.43 (0.31–0.61)	<0.001
T allele	Additive			0.59 (0.48–0.73)	<0.001
Risk genotype					
0–1		156 (32.0)	74 (15.7)	1	
2–3		294 (60.4)	297 (62.9)	2.13 (1.55–2.93)	<0.001
4–5		37 (7.6)	101 (21.4)	5.76 (3.61–9.18)	<0.001

a Adjusted for age, sex, smoking, drinking status, and *Helicobacter pylori* infection status in logistic models.

Given that six polymorphisms showed significant associations with GC risk, we further investigated their joint effects on risk of GC. Because *PSCA* rs2294008 was in 100% LD with *PSCA* rs2976392, we excluded *PSCA* rs2976392 in the following analysis. Compared with subjects carrying 0–1 risk genotypes, individuals carrying 2–3 risk genotypes had an increased risk for GC (OR: 2.13, 95% CI: 1.55–2.93), and those carrying 4–5 risk genotypes had a 4.76‐fold increased risk (OR: 5.76, 95% CI: 3.61–9.18).

The risks of GC related to the polymorphisms were further examined in relation to subtype of GC. We found that the associations of SNPs between controls and noncardia GC cases were similar to those between controls and all GC cases (Table S1). Because there were relatively few cases of cardia GC (74) in our study, the results linking SNPs and risk of cardia GC should be interpreted with caution (Table S2).

### Associations between genetic variants and risk of GC by status of *H. pylori* infection

The main effects of the genetic polymorphisms stratified by type of *H. pylori* infection are shown in Table 3. For *PLCE1* rs2274223 and *PSCA* rs2294008, the effects were mainly present in subjects with Type I *H. pylori* infection. Their adjusted ORs were 1.59 (95% CI: 1.04–2.43) and 1.93 (95% CI: 1.28–2.90), respectively. For *MUC1* rs4072037, similar significant associations between *PRKAA1* rs13361707 and decreased risk of GC were found in subjects with both Types I and II infection. The ORs of *MUC1* rs4072037 in subjects with Types I and II *H. pylori* infection were 0.64 (95% CI: 0.42–0.99) and 0.47 (95% CI: 0.23–0.96), respectively. For *PRKAA1* rs13361707, we found significant associations between *PRKAA1* rs13361707 and decreased risk of GC in subjects with Types I and II infection, and the ORs were 0.46 (95% CI: 0.29–0.73) and 0.39 (95% CI: 0.20–0.78), respectively. Analysis of *ZBTB20* rs9841504 and *SLC52A3* rs13042395 separately did not reveal any effect in subgroups.

**Table 3:** Association between each of the susceptibility loci and risk of GC, stratified by *Helicobacter pylori* infection

*H. pylori* infection	Genotype	Controls *n* (%)	Cases *n* (%)	OR (95% CI)^a^	*P*
*PLCE1* rs2274223					
–	AA	134 (64.7)	34 (60.7)	1.00	
–	AG+GG	73 (35.3)	22 (39.3)	1.59 (0.82–3.09)	0.174
I	AA	111 (68.1)	186 (57.6)	1.00	
I	AG+GG	52 (31.9)	137 (42.4)	1.59 (1.04–2.43)	0.032
II	AA	72 (61.5)	61 (60.4)	1.00	
II	AG+GG	45 (38.5)	40 (39.6)	1.34 (0.74–2.43)	0.328
*PSCA* rs2294008					
–	CC	109 (52.7)	33 (60.0)	1.00	
–	CT+TT	98 (47.3)	22 (40.0)	0.57 (0.28–1.15)	0.116
I	CC	93 (57.1)	133 (41.7)	1.00	
I	CT+TT	70 (42.9)	186 (58.3)	1.93 (1.28–2.90)	0.002
II	CC	65 (55.6)	43 (43.0)	1.00	
II	CT+TT	52 (44.4)	57 (57.0)	1.45 (0.81–2.60)	0.206
*PSCA* rs2976392					
–	GG	109 (52.7)	33 (60.0)	1.00	
–	AA+AG	98 (47.3)	22 (40.0)	0.57 (0.28–1.15)	0.116
I	GG	93 (57.1)	132 (41.8)	1.00	
I	AA+AG	70 (42.9)	184 (58.2)	1.92 (1.27–2.89)	0.002
II	GG	65 (55.6)	42 (42.4)	1.00	
II	AA+AG	52 (44.4)	57 (57.6)	1.50 (0.84–2.69)	0.173
*MUC1* rs4072037					
–	AA	136 (65.7)	45 (81.8)	1.00	
–	AG+GG	71 (34.3)	10 (18.2)	0.44 (0.19–1.01)	0.053
I	AA	101 (62.0)	237 (75.0)	1.00	
I	AG+GG	62 (38.0)	79 (25.0)	0.64 (0.42–0.99)	0.046
II	AA	80 (68.4)	80 (81.6)	1.00	
II	AG+GG	37 (31.6)	18 (18.4)	0.47 (0.23–0.96)	0.037
*ZBTB20* rs9841504					
–	CC	151 (72.9)	45 (80.4)	1.00	
–	CG+GG	56 (27.1)	11 (19.6)	0.57 (0.25–1.33)	0.192
I	CC	115 (70.6)	238 (74.4)	1.00	
I	CG+GG	48 (29.4)	82 (25.6)	0.83 (0.53–1.30)	0.419
II	CC	83 (70.9)	76 (76.0)	1.00	
II	CG+GG	34 (29.1)	24 (24.0)	0.74 (0.38–1.43)	0.371
*SLC52A3* rs13042395					
–	CC	72 (34.6)	25 (44.6)	1.00	
–	CT+TT	136 (65.4)	31 (55.4)	0.76 (0.38–1.49)	0.417
I	CC	67 (41.1)	158 (49.2)	1.00	
I	CT+TT	96 (58.9)	163 (50.8)	0.74 (0.49–1.11)	0.148
II	CC	40 (34.2)	46 (45.5)	1.00	
II	CT+TT	77 (65.8)	55 (54.5)	0.64 (0.35–1.15)	0.137
*PRKAA1* rs13361707					
–	CC	44 (21.4)	21 (38.2)	1.00	
–	CT+TT	162 (78.6)	34 (61.8)	0.50 (0.24–1.04)	0.064
I	CC	34 (20.9)	116 (37.3)	1.00	
I	CT+TT	129 (79.1)	195 (62.7)	0.46 (0.29–0.73)	0.001
II	CC	20 (17.1)	32 (33.3)	1.00	
II	CT+TT	97 (82.9)	64 (66.7)	0.39 (0.20–0.78)	0.007

a Adjusted for age, sex, smoking, and alcohol drinking status in logistic models.

### Effect modification by *H.  pylori* infection status

We investigated the potential interactions between genetic variants and *H. pylori* infection in the risk of GC (Table 4). Significant multiplicative and additive interactions between *PSCA* rs2294008 and *H. pylori* infection on risk of GC were found. The OR due to multiplicative interaction was 3.05 (95% CI: 1.43–6.53) and the SI on an additive interaction scale was 2.45 (95% CI: 1.55–5.52). In contrast, we observed only an additive interaction between *PRKAA1* rs13361707 and *H. pylori* infection on risk of GC (SI on an additive interaction scale: 2.21, 95% CI: 1.39–3.69).

**Table 4:** Joint effects of *Helicobacter pylori* seropositivity and genetic variants on risk of GC

Genotype	*H. pylori* infection	Controls *n* (%)	Cases *n* (%)	OR (95% CI)^a^	*P*
*PLCE1* rs2274223					
AA	−	134 (27.5)	34 (7.1)	1.00	
AA	+	183 (37.6)	247 (51.4)	6.07 (3.72–9.88)	<0.002
AG/GG	−	73 (15.0)	22 (4.6)	1.56 (0.80–3.02)	0.192
AG/GG	+	97 (19.9)	177 (36.9)	8.82 (5.28–14.74)	<0.001
Multiplicative interaction				0.94 (0.45–1.97)	0.861
Additive interaction: SI				1.39 (0.93–2.12)	
*PSCA* rs2294008					
CC	−	109 (22.4)	33 (7.0)	1.00	
CC	+	158 (32.4)	176 (37.1)	3.56 (2.20–5.75)	<0.001
CT/TT	−	98 (20.1)	22 (4.6)	0.57 (0.29–1.14)	0.110
CT/TT	+	122 (25.1)	243 (51.3)	6.20 (3.83–10.05)	<0.001
Multiplicative interaction				3.05 (1.43–6.53)	0.004
Additive interaction: SI				2.45 (1.55–5.52)	
*PSCA* rs2976392					
GG	−	109 (22.4)	33 (7.0)	1.00	
GG	+	158 (32.4)	174 (37.0)	3.51 (2.17–5.69)	<0.001
AG/AA	−	98 (20.1)	22 (4.7)	0.57 (0.29–1.13)	0.110
AG/AA	+	122 (25.1)	241 (51.3)	6.14 (3.79–9.95)	<0.001
Multiplicative interaction				3.06 (1.43–6.54)	0.004
Additive interaction: SI				2.47 (1.43–5.61)	
*MUC1* rs4072037					
AG/GG	−	71 (14.6)	10 (2.1)	1.00	
AG/GG	+	99 (20.3)	97 (20.7)	7.49 (3.38–16.63)	<0.001
AA	−	136 (27.9)	45 (9.6)	2.19 (0.95–5.02)	0.065
AA	+	181 (37.2)	317 (67.6)	12.19 (5.67–26.21)	<0.001
Multiplicative interaction				0.74 (0.30–1.83)	0.520
Additive interaction: SI				1.46 (0.97–2.33)	
*ZBTB20* rs9841504					
CG/GG	−	56 (11.5)	11 (2.3)	1.00	
CG/GG	+	82 (16.8)	106 (22.3)	7.97 (3.54–17.93)	<0.001
CC	−	151 (31.0)	45 (9.4)	1.84 (0.80–4.23)	0.154
CC	+	198 (40.7)	314 (66.0)	9.87 (4.55–21.43)	<0.001
Multiplicative interaction				0.68 (0.27–1.68)	0.397
Additive interaction: SI				1.14 (0.79–1.76)	
*SLC52A3* rs13042395					
CT/TT	−	136 (27.9)	31 (6.5)	1.00	
CT/TT	+	173 (35.4)	218 (45.6)	5.67 (3.51–9.15)	<0.001
CC	−	72 (14.8)	25 (5.2)	1.32 (0.67–2.60)	0.423
CC	+	107 (21.9)	204 (42.7)	8.32 (5.08–13.65)	<0.001
Multiplicative interaction				1.11 (0.52–2.36)	0.781
Additive interaction: SI				1.47 (0.99–2.24)	
*PRKAA1* rs13361707					
CT/TT	−	162 (33.3)	34 (7.4)	1.00	
CT/TT	+	226 (46.5)	259 (56.1)	5.47 (3.51–8.55)	<0.001
CC	−	44 (9.1)	21 (4.5)	2.00 (0.97–4.12)	0.061
CC	+	54 (11.1)	148 (32.0)	13.11 (7.80–22.04)	<0.001
Multiplicative interaction				1.20 (0.53–2.71)	0.663
Additive interaction: SI				2.21 (1.39–3.69)	

a Adjusted for age, sex, smoking, and drinking status in logistic models.

### Associations between the genetic variants and risk of GC by age, gender, smoking, and alcohol drinking status

The main effects of the genetic polymorphisms stratified by age, gender, smoking and alcohol drinking status are shown in Tables S3–S6 *PSCA* rs2294008, *PSCA* rs2976392, *MUC1* rs4072037, and *SLC52A3* rs13042395 were associated with GC risk in subjects who were at young age, male, or smoking. In contrast, for *PRKAA1* rs13361707, significant associations were shown in the subjects irrespective of the age groups, sex, smoking, or drinking status.

### Effect modification by smoking and alcohol drinking status

We did not find any significant interactions between the genetic variants and smoking on risk of GC either in a multiplicative model or on an additive scale (data not shown). However, in the gene–alcohol drinking interaction analysis, there was a significant additive interaction between alcohol drinking and *SLC52A3* rs13042395 in risk of GC, and the SI was 2.13 (95% CI: 1.04–5.73) (Table 5).

**Table 5:** Joint effects of alcohol drinking status and genetic variants on risk of GC

Genotype	Alcohol drinking	Controls *n* (%)	Cases *n* (%)	OR (95% CI)^a^	*P*
*PLCE1* rs2274223					
AA	−	240 (49.2)	134 (32.5)	1.00	
AA	+	77 (15.8)	101 (24.5)	2.83 (1.81–4.42)	<0.001
AG/GG	−	130 (26.6)	95 (23.1)	1.35 (0.94–1.95)	0.106
AG/GG	+	41 (8.4)	82 (19.9)	4.96 (2.98–8.26)	<0.001
Multiplicative interaction				1.30 (0.69–2.45)	0.421
Additive interaction: SI				1.82 (0.89–3.87)	
*PSCA* rs2294008					
CC	−	207 (42.4)	107 (26.3)	1.00	
CC	+	61 (12.5)	77 (18.9)	2.86 (1.74–4.70)	<0.001
CT/TT	−	163 (33.4)	119 (29.2)	1.33 (0.93–1.89)	0.122
CT/TT	+	57 (11.7)	104 (25.6)	4.57 (2.82–7.41)	<0.001
Multiplicative interaction				1.21 (0.65–2.24)	0.555
Additive interaction: SI				1.63 (0.83–3.70)	
*PSCA* rs2976392					
GG	−	207 (42.4)	106 (26.3)	1.00	
GG	+	61 (12.5)	76 (18.8)	2.85 (1.74–4.69)	<0.001
AG/AA	−	163 (33.4)	118 (29.3)	1.32 (0.93–1.89)	0.125
AG/AA	+	57 (11.7)	103 (25.6)	4.57 (2.82–7.41)	<0.001
Multiplicative interaction				1.21 (0.65–2.26)	0.545
Additive interaction: SI				1.64 (0.83–3.83)	
*MUC1* rs4072037					
AG/GG	−	130 (26.6)	55 (13.7)	1.00	
AG/GG	+	40 (8.2)	40 (10.0)	2.95 (1.59–5.49)	0.001
AA	−	240 (49.2)	169 (42.0)	1.67 (1.12–2.49)	0.011
AA	+	78 (16.0)	138 (34.3)	5.26 (3.21–8.63)	<0.001
Multiplicative interaction				1.07 (0.54–2.12)	0.857
Additive interaction: SI				1.62 (0.88–3.66)	
*ZBTB20* rs9841504					
CG/GG	−	101 (20.7)	51 (12.5)	1.00	
CG/GG	+	38 (7.8)	51 (12.5)	3.16 (1.72–5.79)	<0.001
CC	−	269 (55.1)	177 (43.4)	1.31 (0.87–1.98)	0.194
CC	+	80 (16.4)	129 (31.6)	4.23 (2.52–7.09)	<0.001
Multiplicative interaction				1.02 (0.52–2.02)	0.955
Additive interaction: SI				1.31 (0.61–3.36)	
*SLC52A3* rs13042395					
CT/TT	−	231 (47.2)	124 (30.3)	1.00	
CT/TT	+	78 (16.0)	86 (21.0)	2.63 (1.67–4.14)	<0.001
CC	−	139 (28.4)	104 (25.4)	1.25 (0.87–1.80)	0.221
CC	+	41 (8.4)	95 (23.3)	5.01 (3.02–8.31)	<0.001
Multiplicative interaction				1.52 (0.81–2.86)	0.192^b^
Additive interaction: SI				2.13 (1.04–5.73)	
*PRKAA1* rs13361707					
CT/TT	−	296 (60.8)	141 (35.5)	1.00	
CT/TT	+	93 (19.1)	113 (28.5)	3.33 (2.19–5.10)	<0.001
CC	−	74 (15.2)	81 (20.4)	2.46 (1.64–3.68)	<0.001
CC	+	24 (4.9)	62 (15.6)	6.67 (3.70–12.03)	<0.001
Multiplicative interaction				0.82 (0.40–1.67)	0.577
Additive interaction: SI				1.50 (0.73–3.00)	

a Adjusted for age, sex, smoking status, and *Helicobacter pylori* infection status in logistic regression models.

### Association of the seven SNPs with risk of severe IM/dysplasia

We further performed associations between seven genetic variants and risk of severe IM/dysplasia (Table 6). The *MUC1* rs4072037 AG/GG genotype was associated with a decreased risk of **s**evere IM/dysplasia, with an OR of 0.65 (95% CI: 0.46–0.92). We found that the *ZBTB20* rs9841504, CG/GG genotype was associated with a protective effect against risk of severe IM/dysplasia, with an adjusted OR of 0.69 (95% CI: 0.48–0.99). In addition, subjects with a *PRKAA1* rs13361707 CT/TT genotype had a decreased risk of severe IM/dysplasia, with an adjusted OR of 0.68 (95% CI: 0.47–0.98).

**Table 6:** Association of the seven SNPs with risk of severe IM/dysplasia

Genotype	Genetic model	Controls *n* (%)	Severe IM/Dysplasia n (%)	OR (95% CI)^a^	*P* a
*PLCE1* rs2274223					
AA	Codominant	317 (65.0)	197 (70.9)	1	
AG		153 (31.3)	74 (26.6)	0.78 (0.55–1.11)	0.171
GG		18 (3.7)	7 (2.5)	0.67 (0.26–1.71)	0.400
AA	Dominant	317 (65.0)	197 (70.9)	1	
AG+GG		171 (35.0)	81 (29.1)	0.77 (0.55–1.08)	0.133
G allele	Additive			0.80 (0.59–1.07)	0.127
*PSCA* rs2294008					
CC	Codominant	268 (54.9)	146 (52.5)	1	
CT		173 (35.5)	115 (41.4)	1.23 (0.89–1.71)	0.212
TT		47 (9.6)	17 (6.1)	0.74 (0.40–1.37)	0.333
CC	Dominant	268 (54.9)	146 (52.5)	1	
CT+TT		220 (45.1)	132 (47.5)	1.13 (0.83–1.54)	0.440
T allele	Additive			1.01 (0.79–1.28)	0.955
*PSCA* rs2976392					
GG	Codominant	268 (54.9)	146 (52.7)	1	
AG		173 (35.5)	114 (41.2)	1.21 (0.87–1.69)	0.247
AA		47 (9.6)	17 (6.1)	0.74 (0.40–1.36)	0.328
GG	Dominant	268 (54.9)	146 (52.7)	1	
AA+AG		220 (45.1)	131 (47.3)	1.12 (0.82–1.53)	0.492
A allele	Additive			1.00 (0.78–1.27)	0.996
*MUC1* rs4072037					
AA	Codominant	318 (65.2)	207 (74.5)	1	
AG		152 (31.1)	63 (22.6)	0.64 (0.45–0.92)	0.016
GG		18 (3.7)	8 (2.9)	0.70 (0.28–1.76)	0.441
AA	Dominant	318 (65.2)	207 (74.5)	1	
AG+GG		170 (34.8)	71 (25.5)	0.65 (0.46–0.92)	0.014
G allele	Additive			0.70 (0.52–0.95)	0.022
*ZBTB20* rs9841504					
CC	Codominant	349 (71.5)	214 (77.3)	1	
CG		124 (25.4)	48 (17.3)	0.59 (0.40–0.87)	0.008
GG		15 (3.1)	15 (5.4)	1.51 (0.69–3.29)	0.298
CC	Dominant	349 (71.5)	214 (77.3)	1	
CG+GG		139 (28.5)	63 (22.7)	0.69 (0.48–0.99)	0.043
G allele	Additive			0.84 (0.63–1.13)	0.245
*SLC52A3* rs13042395					
CC	Codominant	180 (36.8)	117 (42.1)	1	
CT		247 (50.5)	134 (48.2)	0.89 (0.64–1.23)	0.476
TT		62 (12.7)	27 (9.7)	0.58 (0.34–0.99)	0.045
CC	Dominant	180 (36.8)	117 (42.1)	1	
CT+TT		309 (63.2)	161 (57.9)	0.82 (0.60–1.12)	0.215
T allele	Additive			0.80 (0.63–1.02)	0.067
*PRKAA1* rs13361707					
CC	Codominant	98 (20.1)	71 (25.8)	1	
CT		246 (50.5)	139 (50.6)	0.71 (0.48–1.04)	0.081
TT		143 (29.4)	65 (23.6)	0.62 (0.40–0.97)	0.037
CC	Dominant	98 (20.1)	71 (25.8)	1	
CT+TT		389 (79.9)	204 (74.2)	0.68 (0.47–0.98)	0.038
T allele	Additive			0.79 (0.63–0.99)	0.040

a Adjusted for age, sex, smoking, and alcohol drinking status and *Helicobacter pylori* infection status in logistic regression models.

## Discussion

In this study, we covered all seven GWAS‐identified GC susceptibility loci and explored their potential interactions with environmental factors (*H. pylori* infection, smoking and alcohol drinking) on risk of GC. We further examined their potential effects on risk of precancerous lesions of the stomach. We confirmed that *PLCE1* rs2274223, *PSCA* rs2294008, *PSCA* rs2976392, *MUC1* rs4072037, *SCL52A3* rs13042395 and *PRKAA1* rs13361707 were associated with GC risk in a Chinese population. The risk of GC may be modulated by *PSCA* rs2294008 and *PRKAA1* rs13361707, possibly in combination with *H. pylori* infection. Meanwhile, *SCL52A3* rs13042395 had a potential interaction with alcohol drinking and had an effect on risk of GC. Moreover, three SNPs, *MUC1* rs4072037, *ZBTB20* rs9841504, and *PRAKK1* rs13361707, were also associated with gastric severe IM/dysplasia.

Studies on interactions between these seven genetic polymorphisms and *H. pylori* infection on risk of GC are limited and yielded inconsistent results 20, 32. This inconsistency may be due to variations in study populations with sample sizes. Previous reports from Japanese and Caucasians 32, 33 found that individuals who had *H. pylori* infection and a *PSCA* risk genotype might have a higher risk of GC than subjects without *H. pylori* infection, consistent with our results. However, these authors did not find significant interactions in their study, perhaps due to the small sample sizes in their studies. In our study, we found significant interactions between the *PSCA* polymorphism and *H. pylori* infection on risk of GC. An individual who carries the *PSCA* risk genotype and Type I *H. pylori* infection may have a higher risk of GC than an individual with Type II infection. PSCA, a tumor suppressor, is thought to have an inhibitory effect on the proliferation of differentiating gastric epithelial cells, and the downregulation of *PSCA* expression can be found in GC tissues 34. The T allele of *PSCA* rs2294008 may reduce the transcriptional activity of the *PSCA* promoter in gastric cell lines 13. Type I *H. pylori* strain, expressing CagA and/or VacA, release proinflammatory cytokines and induce vacuole formation, which results in higher toxicity than Type II strain 8, 10. Studies indicate that the *PSCA* genotype may be related in some way to parietal cell mass or to the regulation of gastric acid secretion, which then may influence the effects of *H. pylori* ‐associated inflammation 35. The different pathogenicity of the two types of *H. pylori* and their joint effect with *PSCA* genotype may play a part of process of inflammatory‐related gastric carcinogenesis, which makes the individual who carries *PSCA* risk genotype and Type I *H. pylori* infection have a higher risk of GC. We found significant interactions between *PRKAA1* rs13361707 and *H. pylori* infection in risk of GC. Eom et al. 36 found an additive interaction between *H. pylori* CagA infection and *PRKAA1* polymorphism, and our results are also consistent with their findings (data not shown). We further identified that in subjects with Types I and II *H. pylori* infection, there were significant associations between *PRKAA1* polymorphisms and GC risk.

*SLC52A3* rs13042395 is located on chromosome 20p13. This gene encodes riboflavin transporter 2 protein (RFT2), which plays a role in gastric carcinogenesis 37. A previous study conducted by Dong *et al*. 38 found that subjects carrying *SLC52A3* rs13042395 T carriers showed a significantly increased risk of noncardia GC when they were alcohol drinkers, similar to the results of our study. In addition, we identified an additive interaction between *SLC52A3* rs13042395 and alcohol drinking status, suggesting that *SLC52A3* may interact with certain metabolic changes induced by alcohol drinking and influence the susceptibility to GC.

GC is an end result of the transformation of multistage precancerous gastric lesions, including chronic atrophic gastritis, IM, and dysplasia. Previous studies have found that certain genetic variants associated with GC, such as *PSCA* rs2294008 may also have an effect on risk of precancerous gastric lesions, though the results were inconsistent 23, 39, potentially due to different sample sizes and population heterogeneity. In our study, we identified that *MUC1* rs4072037 and *PRKAA1* rs13361707, which were significantly associated with GC, and *ZBTB20* rs9841504, which was not, were also related to severe IM/dysplasia, indicating that these polymorphisms may affect an early stage of gastric carcinogenesis.

One of the strengths of our study is that we collected detailed personal information regarding environmental exposures from the study subjects, which enabled us to estimate the effect modification of environmental factors. Moreover, the severe IM/dysplasia was diagnosed from the same screening population as the control subjects, allowing us to explore the effects of seven susceptibility loci on the risk of precancerous lesions as well as GC. However, there are some limitations in our study. First, although we had a total of 1273 subjects in our study, the sample size was not sufficiently large for stratification analysis. Due to the multiple comparisons performed, false‐positive results might have occurred. However, after multiple test comparison correction using the Benjamini–Hochberg procedure, we were still able to observe significant independent and interactive associations of *H. pylori* infection and genetic polymorphisms on GC risk. Future large‐scale studies and functional studies will be needed to further validate our results.

In summary, we systematically explored the seven GWAS‐identified susceptibility loci and their potential interactions with environmental factors in risk of GC. We found that *PSCA* and *PRKAA1* polymorphisms may interact with *H. pylori* infection and that they had a synergistic effect on risk of GC. Meanwhile, *SLC52A3* rs13042395 had an additive interaction with alcohol drinking that may contribute to GC risk. Three genetic variants that were associated with risk of GC may also be related to risk of severe IM/dysplasia. Knowledge of gene–environment interactions is important for risk prediction and the identification of certain high‐risk populations, which in turn informs public health strategies for targeted prevention. Our study may also be helpful for gaining insight into the biological mechanisms underlying the associations between specific risk factors and gastric carcinogenesis. Additional studies with larger cohorts, as well as mechanistic studies of these findings are warranted.

## Conflict of Interest

We have no conflict of interest to declare.

## Supporting information


**Table S1.** Association of the seven SNPs with risk of noncardia cases and controls.
**Table S2.** Association of the seven SNPs with risk of cardia GC cases and controls.
**Table S3.** Association between each of the susceptibility loci and risk of GC, stratified by age.
**Table S4.** Association between each of the susceptibility loci and risk of GC, stratified by sex.
**Table S5.** Association between each of the susceptibility loci and risk of GC, stratified by smoking status.
**Table S6.** Association between each of the susceptibility loci and risk of GC, stratified by alcohol drinking status.
