# Supplementary material for: Environmental factors, seven GWAS‐identified susceptibility loci, and risk of gastric cancer and its precursors in a Chinese population
Source: Cancer Med. 2017 Feb 21;6(3):708–20. doi: 10.1002/cam4.1038 (PMC5345626; doi:10.1002/cam4.1038)
Supplement: Supplementary file 1 — Table S1. Association of the seven SNPs with risk of noncardia cases and controls. Table S2. Association of the seven SNPs with risk of cardia GC cases and controls. Table S3. Association between each of the susceptibility loci and risk of GC, stratified by age. Table S4. Association between each of the susceptibility loci and risk of GC, stratified by sex. Table S5. Association between each of the susceptibility loci and risk of GC, stratified by smoking status. Table S6. Association between each of the susceptibility loci and risk of GC, stratified by alcohol drinking status. [file CAM4-6-708-s001.doc]

**Environmental factors, seven GWAS-identified susceptibility loci and risk of gastric cancer and its precursors in a Chinese population**

Meng Cai^1^, Shuyang Dai^1^, Wanqing Chen^2^, Changfa Xia^2^, Lingeng Lu^3^, Shuguang Dai^4^, Jun Qi^5^, Minjie Wang^5^, Meilin Wang^6, 7^, Lanping Zhou^1^, Fuhua Lei^8^, Tingting Zuo^2^, Hongmei Zeng^2, †^, Xiaohang Zhao^1, †^

^1^State Key Laboratory of Molecular Oncology, National Cancer Center/Cancer Hospital, Chinese Academy of Medical Sciences and Peking Union Medical College, Beijing, China.

^2^National Office for Cancer Prevention and Control, National Cancer Center/Cancer Hospital, Chinese Academy of Medical Sciences and Peking Union Medical College, Beijing, China.

^3^Department of Chronic Disease Epidemiology, Yale School of Public Health, School of Medicine, Yale Cancer Center, Yale University, New Haven, USA.

^4^Center for Disease Control and Prevention of Sheyang County, Jiangsu Province, China.

^5^Department of Clinical Laboratory, National Cancer Center/Cancer Hospital, Chinese Academy of Medical Sciences and Peking Union Medical College, Beijing, China.

^6^Department of Environmental Genomics, Jiangsu Key Laboratory of Cancer Biomarkers, Prevention and Treatment, Collaborative Innovation Center for Cancer Personalized Medicine, Nanjing Medical University, Nanjing, China.

^7^Department of Genetic Toxicology, The Key Laboratory of Modern Toxicology of Ministry of Education, School of Public Health, Nanjing Medical University, Nanjing, China.

^8^Department of Pathology, Feicheng People Hospital, Shandong Province, China.

**Supporting Information**

Additional supporting information may be found in the online version of this article:

**Table S1.** Association of the seven SNPs with risk of Non-cardia cases and controls.

**Table S2.** Association of the seven SNPs with risk of cardia GC cases and controls.

**Table S3.** Association between each of the susceptibility loci and risk of GC, stratified by age.

**Table S4.** Association between each of the susceptibility loci and risk of GC, stratified by sex.

**Table S5.** Association between each of the susceptibility loci and risk of GC, stratified by smoking status.

**Table S6.** Association between each of the susceptibility loci and risk of GC, stratified by alcohol drinking status.

**Supplementary Materials**

**Supplemental Table 1.** Association of the seven SNPs with risk of Non-cardia cases and controls.

| Genotype | Genetic Model | Controls n(%) | Non-cardia cases n(%) | OR(95%CI) ^a^ | *P* |
| --- | --- | --- | --- | --- | --- |
| ***PLCE1* rs2274223** |  |  |  |  |  |
| AA | Codominant | 317(65.0) | 219(57.9) | 1.00 |  |
| AG |  | 153(31.3) | 138(36.5) | 1.36(0.98-1.88) | 0.067 |
| GG |  | 18(3.7) | 21(5.6) | 2.33(1.10-4.92) | 0.027 |
| AA | Dominant | 317(65.0) | 219(57.9) | 1.00 |  |
| AG+GG |  | 171(35.0) | 159(42.1) | 1.44(1.05-1.98) | 0.022 |
| G Allele | Additive |  |  | 1.43(1.09-1.86) | 0.009 |
| ***PSCA* rs2294008** |  |  |  |  |  |
| CC | Codominant | 268(54.9) | 171(45.8） | 1.00 |  |
| CT |  | 173(35.5) | 170(45.6) | 1.42(1.02-1.96) | 0.036 |
| TT |  | 47(9.6) | 32(8.6) | 0.98(0.56-1.71) | 0.948 |
| CC | Dominant | 268(54.9) | 171(45.8） | 1.00 |  |
| CT+TT |  | 220(45.1) | 202(54.2) | 1.32(0.97-1.80) | 0.076 |
| T Allele | Additive |  |  | 1.14(0.90-1.44) | 0.282 |
| ***PSCA* rs2976392** |  |  |  |  |  |
| GG | Codominant | 268(54.9) | 170(45.9) | 1.00 |  |
| AG |  | 173(35.5) | 168(45.4) | 1.40(1.01-1.95) | 0.042 |
| AA |  | 47(9.6) | 32(8.7) | 0.99(0.57-1.72) | 0.963 |
| GG | Dominant | 268(54.9) | 170(45.9) | 1.00 |  |
| AA+AG |  | 220(45.1) | 200(54.1) | 1.31(0.96-1.79) | 0.084 |
| A Allele | Additive |  |  | 1.14(0.90-1.44) | 0.291 |
| ***MUC1* rs4072037** |  |  |  |  |  |
| AA | Codominant | 318(65.2) | 284(77.0) | 1.00 |  |
| AG |  | 152(31.1) | 71(19.2) | 0.57(0.40-0.82) | 0.002 |
| GG |  | 18(3.7) | 14(3.8) | 1.13(0.51-2.50) | 0.762 |
| AA | Dominant | 318(65.2) | 284(77.0) | 1.00 |  |
| AG+GG |  | 170(34.8) | 85(23.0) | 0.63(0.44-0.88) | 0.007 |
| G Allele | Additive |  |  | 0.75(0.56-0.99) | 0.045 |
| ***ZBTB20* rs9841504** |  |  |  |  |  |
| CC | Codominant | 349(71.5) | 282(75.2) | 1.00 |  |
| CG |  | 124(25.4) | 74(19.7) | 0.66(0.46-0.96) | 0.028 |
| GG |  | 15(3.1) | 19(5.1) | 1.71(0.77-3.81) | 0.188 |
| CC | Dominant | 349(71.5) | 282(75.2) | 1.00 |  |
| CG+GG |  | 139(28.5) | 93(24.8) | 0.76(0.54-1.07) | 0.118 |
| G Allele | Additive |  |  | 0.90(0.68-1.20) | 0.467 |
| ***SLC52A3* rs13042395** |  |  |  |  |  |
| CC | Codominant | 180(36.8) | 189(50.4) | 1.00 |  |
| CT |  | 247(50.5) | 158(42.1) | 0.73(0.53-1.01) | 0.057 |
| TT |  | 62(12.7) | 28(7.5) | 0.41(0.24-0.71) | 0.002 |
| CC | Dominant | 180(36.8) | 189(50.4) | 1.00 |  |
| CT+TT |  | 309(63.2) | 186(49.6) | 0.66(0.48-0.90) | 0.008 |
| T Allele | Additive |  |  | 0.67(0.53-0.85) | 0.001 |
| ***PRKAA1* rs13361707** |  |  |  |  |  |
| CC | Codominant | 98(20.1) | 132(36.4) | 1.00 |  |
| CT |  | 246(50.5) | 167(46.0) | 0.51(0.35-0.74) | <0.001 |
| TT |  | 143(29.4) | 64(17.6) | 0.34(0.22-0.54) | <0.001 |
| CC | Dominant | 98(20.1) | 132(36.4) | 1.00 |  |
| CT+TT |  | 389(79.9) | 231(63.6) | 0.45(0.32-0.64） | <0.001 |
| T Allele | Additive |  |  | 0.58(0.46-0.73) | <0.001 |

a. Adjusted for age, sex, smoking, alcohol drinking status and *H. pylori* infection status in logistic regression models.

**Supplemental Table 2.** Association of the seven SNPs with risk of cardia GC cases and controls.

| Genotype | Genetic Model | Controls  n (%) | Cardia cases  n (%) | OR (95% CI) ^a^ | *P* |
| --- | --- | --- | --- | --- | --- |
| ***PLCE1* rs2274223** |  |  |  |  |  |
| AA | Codominant | 317(65.0) | 39(55.0) | 1.00 |  |
| AG |  | 153(31.3) | 27(38.0) | 2.01(1.05-3.83) | 0.035 |
| GG |  | 18(3.7) | 5(7.0) | 3.24(0.98-10.67) | 0.054 |
| AA | Dominant | 317(65.0) | 39(55.0) | 1.00 |  |
| AG+GG |  | 171(35.0) | 32(45.0) | 2.15(1.16-3.99) | 0.015 |
| G Allele | Additive |  |  | 1.89(1.16-3.07) | 0.010 |
| ***PSCA* rs2294008** |  |  |  |  |  |
| CC | Codominant | 268(54.9) | 26(36.6) | 1.00 |  |
| CT |  | 173(35.5) | 36(50.7) | 2.34(1.20-4.54) | 0.012 |
| TT |  | 47(9.6) | 9(12.7) | 2.03(0.74-5.55) | 0.168 |
| CC | Dominant | 268(54.9) | 26(36.6) | 1.00 |  |
| CT+TT |  | 220(45.1) | 45(63.4) | 2.27(1.20-4.29) | 0.012 |
| T Allele | Additive |  |  | 1.62(1.05-2.51) | 0.031 |
| ***PSCA* rs2976392** |  |  |  |  |  |
| GG | Codominant | 268(54.9) | 26(36.6) | 1.00 |  |
| AG |  | 173(35.5) | 36(50.7) | 2.34(1.20-4.54) | 0.012 |
| AA |  | 47(9.6) | 9(12.7) | 2.03(0.74-5.55) | 0.168 |
| GG | Dominant | 268(54.9) | 26(36.6) | 1.00 |  |
| AA+AG |  | 220(45.1) | 45(63.4) | 2.27(1.20-4.29) | 0.012 |
| A Allele | Additive |  |  | 1.62(1.05-2.51) | 0.031 |
| ***MUC1* rs4072037** |  |  |  |  |  |
| AA | Codominant | 318(65.2) | 56(78.9) | 1.00 |  |
| AG |  | 152(31.1) | 14(19.7) | 0.40(0.18-0.89) | 0.024 |
| GG |  | 18(3.7) | 1(1.4) | 0.48(0.06-3.99) | 0.498 |
| AA | Dominant | 318(65.2) | 56(78.9) | 1.00 |  |
| AG+GG |  | 170(34.8) | 15(21.1) | 0.41(0.19-0.87) | 0.021 |
| G Allele | Additive |  |  | 0.47(0.23-0.93) | 0.030 |
| ***ZBTB20* rs9841504** |  |  |  |  |  |
| CC | Codominant | 349(71.5) | 53(75.7) | 1.00 |  |
| CG |  | 124(25.4) | 13(18.6) | 0.68(0.32-1.47) | 0.332 |
| GG |  | 15(3.1) | 4(5.7) | 2.12(0.52-8.69) | 0.295 |
| CC | Dominant | 349(71.5) | 53(75.7) | 1.00 |  |
| CG+GG |  | 139(28.5) | 17(24.3) | 0.82(0.41-1.64) | 0.569 |
| G Allele | Additive |  |  | 0.98(0.55-1.74) | 0.931 |
| ***SLC52A3* rs13042395** |  |  |  |  |  |
| CC | Codominant | 180(36.8) | 33(45.8) | 1.00 |  |
| CT |  | 247(50.5) | 35(48.6) | 0.74(0.40-1.40) | 0.355 |
| TT |  | 62(12.7) | 4(5.6) | 0.18(0.04-0.83) | 0.027 |
| CC | Dominant | 180(36.8) | 33(45.8) | 1.00 |  |
| CT+TT |  | 309(63.2) | 39(54.2) | 0.60(0.33-1.11) | 0.107 |
| T Allele | Additive |  |  | 0.57(0.35-0.93) | 0.024 |
| ***PRKAA1* rs13361707** |  |  |  |  |  |
| CC | Codominant | 98(20.1) | 19(27.1) | 1.00 |  |
| CT |  | 246(50.5) | 32(45.7) | 0.52(0.25-1.10) | 0.089 |
| TT |  | 143(29.4) | 19(27.2) | 0.57(0.25-1.33) | 0.193 |
| CC | Dominant | 98(20.1) | 19(27.1) | 1.00 |  |
| CT+TT |  | 389(79.9) | 51(72.9) | 0.54(0.27-1.08) | 0.082 |
| T Allele | Additive |  |  | 0.75(0.48-1.17) | 0.211 |

a. Adjusted for age, sex, smoking, alcohol drinking status and *H. pylori* infection status in logistic regression models.

**Supplemental Table 3.** Association between each of the susceptibility loci and risk of GC, stratified by age.

| Genotype | Age | Controls n (%) | Cases n (%) | OR (95%CI) ^a^ | *P* |
| --- | --- | --- | --- | --- | --- |
| ***PLCE1* rs2274223** |  |  |  |  |  |
| AA | <58 | 154(66.4) | 151(62.1) | 1 |  |
| AG+GG |  | 78(33.6) | 92(37.9) | 1.48(0.95-2.31) | 0.081 |
| AA | ≥58 | 163(63.7) | 140(56.5) | 1 |  |
| AG+GG |  | 93(36.3) | 108(43.5) | 1.50(0.99-2.26) | 0.055 |
| ***PSCA* rs2294008** |  |  |  |  |  |
| CC | <58 | 136(58.6) | 103(43.1) | 1 |  |
| CT+TT |  | 96(41.4) | 136(56.9) | 1.65(1.07-2.54) | 0.022 |
| CC | ≥58 | 132(51.6) | 112(45.5) | 1 |  |
| CT+TT |  | 124(48.4) | 134(54.5) | 1.24(0.83-1.86) | 0.291 |
| ***PSCA* rs2976392** |  |  |  |  |  |
| GG | <58 | 136(58.6) | 101(42.8) | 1 |  |
| AA+AG |  | 96(41.4) | 135(57.2) | 1.68(1.09-2.58) | 0.019 |
| GG | ≥58 | 132(51.6) | 112(45.7) | 1 |  |
| AA+AG |  | 124(48.4) | 133(54.3) | 1.23(0.82-1.84) | 0.317 |
| ***MUC1* rs4072037** |  |  |  |  |  |
| AA | <58 | 147(63.4) | 185(78.4) | 1 |  |
| AG+GG |  | 85(36.6) | 51(21.6) | 0.51(0.32-0.83) | 0.007 |
| AA | ≥58 | 171(66.8) | 186(76.2) | 1 |  |
| AG+GG |  | 85(33.2) | 58(23.8) | 0.65(0.42-1.02) | 0.059 |
| ***ZBTB20* rs9841504** |  |  |  |  |  |
| CC | <58 | 166(71.6) | 177(73.8) | 1 |  |
| CG+GG |  | 66(28.4) | 63(26.2) | 0.73(0.45-1.17) | 0.191 |
| CC | ≥58 | 183(71.5) | 191(77.3) | 1 |  |
| CG+GG |  | 73(28.5) | 56(22.7) | 0.76(0.48-1.21) | 0.248 |
| ***SLC52A3* rs13042395** |  |  |  |  |  |
| CC | <58 | 82(35.2) | 123(50.8) | 1 |  |
| CT+TT |  | 151(64.8) | 119(49.2) | 0.61(0.40-0.94) | 0.026 |
| CC | ≥58 | 98(38.3) | 115(46.6) | 1 |  |
| CT+TT |  | 158(61.7) | 132(53.4) | 0.82(0.54-1.23) | 0.336 |
| ***PRKAA1* rs13361707** |  |  |  |  |  |
| CC | <58 | 48(20.7) | 90(39.0) | 1 |  |
| CT+TT |  | 184(79.3) | 141(61.0) | 0.44(0.27-0.71) | 0.001 |
| CC | ≥58 | 50(19.6) | 82(33.9) | 1 |  |
| CT+TT |  | 205(80.4) | 160(66.1) | 0.44(0.27-0.71) | 0.001 |
| a. Adjusted for *H. pylori* infection, sex, smoking and alcohol drinking status in logistic regression models. | | | | | |

| **Supplemental Table 4.** Association between each of the susceptibility loci and risk of GC, stratified by sex. | | | | | |
| --- | --- | --- | --- | --- | --- |
| Genotype | Sex | Controls n (%) | Cases n (%) | OR (95%CI) ^a^ | *P* |
| ***PLCE1* rs2274223** |  |  |  |  |  |
| AA | male | 224(63.8) | 212(59.6) | 1 |  |
| AG+GG |  | 127(36.2) | 144(40.4) | 1.41(0.99-2.01) | 0.056 |
| AA | female | 93(67.9) | 79(58.5) | 1 |  |
| AG+GG |  | 44(32.1) | 56(41.5) | 1.64(0.94-2.89) | 0.084 |
| ***PSCA* rs2294008** |  |  |  |  |  |
| CC | male | 191(54.4) | 146(41.5) | 1 |  |
| CT+TT |  | 160(45.6) | 206(58.5) | 1.60(1.13-2.26) | 0.009 |
| CC | female | 77(56.2) | 69(51.9) | 1 |  |
| CT+TT |  | 60(43.8) | 64(48.1) | 1.02(0.59-1.76) | 0.949 |
| ***PSCA* rs2976392** |  |  |  |  |  |
| GG | male | 191(54.4) | 145(41.5) | 1 |  |
| AA+AG |  | 160(45.6) | 204(58.5) | 1.58(1.12-2.24) | 0.010 |
| GG | female | 77(56.2) | 68(51.5) | 1 |  |
| AA+AG |  | 60(43.8) | 64(48.5) | 1.03(0.60-1.78) | 0.906 |
| ***MUC1* rs4072037** |  |  |  |  |  |
| AA | male | 222(63.2) | 266(76.4) | 1 |  |
| AG+GG |  | 129(36.8) | 82(23.6) | 0.57(0.39-0.84) | 0.004 |
| AA | female | 96(70.1) | 105(79.5) | 1 |  |
| AG+GG |  | 41(29.9) | 27(20.5) | 0.61(0.33-1.12) | 0.110 |
| ***ZBTB20* rs9841504** |  |  |  |  |  |
| CC | male | 254(72.4) | 265(75.1) | 1 |  |
| CG+GG |  | 97(27.6) | 88(24.9) | 0.73(0.50-1.08) | 0.117 |
| CC | female | 95(69.3) | 103(76.9) | 1 |  |
| CG+GG |  | 42(30.7) | 31(23.1) | 0.84(0.45-1.55) | 0.565 |
| ***SLC52A3* rs13042395** |  |  |  |  |  |
| CC | male | 128(36.4) | 172(48.6) | 1 |  |
| CT+TT |  | 224(63.6) | 182(51.4) | 0.72(0.51-1.02) | 0.063 |
| CC | female | 52(38.0) | 66(48.9) | 1 |  |
| CT+TT |  | 85(62.0) | 69(51.1) | 0.65(0.38-1.12) | 0.120 |
| ***PRKAA1* rs13361707** |  |  |  |  |  |
| CC | male | 73(20.9) | 125(36.4) | 1 |  |
| CT+TT |  | 277(79.1) | 218(63.6) | 0.46(0.31-0.69) | <0.001 |
| CC | female | 25(18.2) | 47(36.2) | 1 |  |
| CT+TT |  | 112(81.8) | 83(63.8) | 0.37(0.20-0.70) | 0.002 |
| a. Adjusted for *H. pylori* infection, age, smoking status and alcohol drinking status in logistic regression models. | | | | | |

| **Supplemental Table 5.** Association between each of the susceptibility loci and risk of GC, stratified by smoking status. | | | | | |
| --- | --- | --- | --- | --- | --- |
| Genotype | Smoking | Controls n (%) | Cases n (%) | OR(95%CI) ^a^ | *P* |
| ***PLCE1* rs2274223** |  |  |  |  |  |
| AA | - | 187(63.8) | 119(53.8) | 1 |  |
| AG+GG |  | 106(36.2) | 102(46.2) | 1.46(0.99-2.14) | 0.054 |
| AA | + | 130(66.7) | 118(60.5) | 1 |  |
| AG+GG |  | 65(33.3) | 77(39.5) | 1.48(0.91-2.41) | 0.113 |
| ***PSCA* rs2294008** |  |  |  |  |  |
| CC | - | 158(53.9) | 105(48.4) | 1 |  |
| CT+TT |  | 135(46.1) | 112(51.6) | 1.15(0.79-1.68) | 0.474 |
| CC | + | 110(56.4) | 80(41.2) | 1 |  |
| CT+TT |  | 85(43.6) | 114(58.8) | 1.97(1.23-3.14) | 0.005 |
| ***PSCA* rs2976392** |  |  |  |  |  |
| GG | - | 158(53.9) | 103(47.9) | 1 |  |
| AA+AG |  | 135(46.1) | 112(52.1) | 1.17(0.80-1.71) | 0.421 |
| GG | + | 110(56.4) | 80(41.7) | 1 |  |
| AA+AG |  | 85(43.6) | 112(58.3) | 1.92(1.20-3.08) | 0.006 |
| ***MUC1* rs4072037** |  |  |  |  |  |
| AA | - | 199(67.9) | 163(76.2) | 1 |  |
| AG+GG |  | 94(32.1) | 51(23.8) | 0.65(0.42-1.00) | 0.048 |
| AA | + | 119(61.0) | 146(76.0) | 1 |  |
| AG+GG |  | 76(39.0) | 46(24.0) | 0.48(0.29-0.80) | 0.005 |
| ***ZBTB20* rs9841504** |  |  |  |  |  |
| CC | - | 211(72.0) | 167(76.3) | 1 |  |
| CG+GG |  | 82(28.0) | 52(23.7) | 0.75(0.49-1.16) | 0.200 |
| CC | + | 138(70.8) | 142(73.6) | 1 |  |
| CG+GG |  | 57(29.2) | 51(26.4) | 0.80(0.48-1.34) | 0.400 |
| ***SLC52A3* rs13042395** |  |  |  |  |  |
| CC | - | 112(38.2) | 103(47.0) | 1 |  |
| CT+TT |  | 181(61.8) | 116(53.0) | 0.78(0.53-1.14) | 0.192 |
| CC | + | 68(34.7) | 96(49.5) | 1 |  |
| CT+TT |  | 128(65.3) | 98(50.5) | 0.59(0.37-0.94) | 0.028 |
| ***PRKAA1* rs13361707** |  |  |  |  |  |
| CC | - | 55(18.8) | 78(36.8) | 1 |  |
| CT+TT |  | 237(81.2) | 134(63.2) | 0.38(0.25-0.59) | <0.001 |
| CC | + | 43(22.1) | 65(34.4) | 1 |  |
| CT+TT |  | 152(77.9) | 124(65.6) | 0.53(0.31-0.90) | 0.019 |
| a. Adjusted for *H. pylori* infection, age, sex and alcohol drinking status in logistic regression models. | | | | | |

| **Supplemental Table 6.** Association between each of the susceptibility loci and risk of GC, stratified by alcohol drinking status. | | | | | |
| --- | --- | --- | --- | --- | --- |
| Genotype | alcohol Drinking | Controls n (%) | Cases n (%) | OR(95%CI) ^a^ | *P* |
| ***PLCE1* rs2274223** |  |  |  |  |  |
| AA | - | 240(64.9) | 134(58.5) | 1 |  |
| AG+GG |  | 130(35.1) | 95(41.5) | 1.46(0.99-2.14) | 0.054 |
| AA | + | 130(66.7) | 118(60.5) | 1 |  |
| AG+GG |  | 65(33.3) | 77(39.5) | 1.29(0.90-1.87) | 0.171 |
| ***PSCA* rs2294008** |  |  |  |  |  |
| CC | - | 207(55.9) | 107(47.3) | 1 |  |
| CT+TT |  | 163(44.1) | 119(52.7) | 1.15(0.79-1.68) | 0.474 |
| CC | + | 110(56.4) | 80(41.2) | 1 |  |
| CT+TT |  | 85(43.6) | 114(58.8) | 1.32(0.92-1.88) | 0.133 |
| ***PSCA* rs2976392** |  |  |  |  |  |
| GG | - | 207(55.9) | 106(47.3) | 1 |  |
| AA+AG |  | 163(44.1) | 118(52.7) | 1.17(0.80-1.71) | 0.421 |
| GG | + | 110(56.4) | 80(41.7) | 1 |  |
| AA+AG |  | 85(43.6) | 112(58.3) | 1.31(0.92-1.88) | 0.136 |
| ***MUC1* rs4072037** |  |  |  |  |  |
| AA | - | 240(64.9) | 169(75.4) | 1 |  |
| AG+GG |  | 130(35.1) | 55(24.6) | 0.65(0.42-1.00) | 0.048 |
| AA | + | 119(61.0) | 146(76.0) | 1 |  |
| AG+GG |  | 76(39.0) | 46(24.0) | 0.59(0.40-0.88) | 0.010 |
| ***ZBTB20* rs9841504** |  |  |  |  |  |
| CC | - | 269(72.7) | 177(77.6) | 1 |  |
| CG+GG |  | 101(27.3) | 51(22.4) | 0.75(0.49-1.16) | 0.200 |
| CC | + | 138(70.8) | 142(73.6) | 1 |  |
| CG+GG |  | 57(29.2) | 51(26.4) | 0.77(0.51-1.16) | 0.210 |
| ***SLC52A3* rs13042395** |  |  |  |  |  |
| CC | - | 139(37.6) | 104(45.6) | 1 |  |
| CT+TT |  | 231(62.4) | 124(54.4) | 0.78(0.53-1.14) | 0.192 |
| CC | + | 68(34.7) | 96(49.5) | 1 |  |
| CT+TT |  | 128(65.3) | 98(50.5) | 0.81(0.56-1.16) | 0.244 |
| ***PRKAA1* rs13361707** |  |  |  |  |  |
| CC | - | 74(20.0) | 81(36.5) | 1 |  |
| CT+TT |  | 296(80.0) | 141(63.5) | 0.38(0.25-0.59) | <0.001 |
| CC | + | 43(22.1) | 65(34.4) | 1 |  |
| CT+TT |  | 152(77.9) | 124(65.6) | 0.40(0.27-0.61) | <0.001 |

a. Adjusted for *H. pylori* infection, age, sex and smoking status in logistic regression models.
